# Use of sarolaner in the treatment of tungiasis in naturally infested dogs

**DOI:** 10.29374/2527-2179.bjvm000224

**Published:** 2024-03-13

**Authors:** Rafaella Tortoriello, Natália Lôres Lopes, Bianca Bianco Paschoalino Linhares, Thais Ribeiro Correia, Julio Israel Fernandes

**Affiliations:** 1 Veterinarian, MSc., Programa de Pós-Graduação em Ciências Veterinárias (PPGCV), Departamento de Parasitologia Animal (DPA) Instituto de Veterinária (IV), Universidade Federal Rural do Rio de Janeiro (UFRRJ), Seropédica, RJ, Brazil.; 2 Veterinarian, DSc., Programa de Pós-Graduação em Medicina Veterinária (PPGMV), Departamento de Medicina e Cirurgia Veterinária (DMCV), UFRRJ, Seropédica, RJ, Brazil.; 3 Veterinarian, Autonomous DERMAPET, Juiz de Fora, MG, Brazil.; 4 Veterinarian, DSc. DPA, IV, UFRRJ, Seropédica, RJ, Brazil.; 5 Veterinarian, DSc. DMCV, IV, UFRRJ, Seropédica, RJ, Brazil.

**Keywords:** Tunga spp., canine, isoxazoline, control, prophylaxis, *Tunga* spp., canino, isoxazolina, controle, profilaxia

## Abstract

Tungiasis is an endemic dermatological parasitic zoonosis in Latin America, caused by the sand flea *Tunga* spp*.* (Siphonaptera, Tungidae), which promotes intense discomfort, swelling, erythema, itching, pain, secondary bacterial infection, cellulitis and necrosis. Sarolaner has been used to control different ectoparasites, but there is no record of its use for the treatment of tungiasis in dogs. The objective of this study was to evaluate the effectiveness of sarolaner for the treatment dogs naturally infested by *Tunga* spp*.* kept in the same infested environment. Three of four animals were medicated with sarolaner orally with a single dose of 2 mg/kg, as recommended by the manufacturer, and one animal remained without medication. After 24 hours, the fleas from all four dogs were mechanically removed. The animals were reevaluated on days +15 and +30 to assess possible reinfestation. The medicated animals remained free of fleas, while the untreated animal had fleas on the days previously defined for reevaluation. We can thus conclude that the use of sarolaner is an effective choice for tungiasis treatment.

## Resumo

Tungíase é uma zoonose parasitária dermatológica endêmica na América Latina, causada pela pulga da areia *Tunga* spp. (Siphonaptera, Tungidae), que promove intenso desconforto, edema, eritema, prurido, dor, infecção bacteriana secundária, celulite e necrose. Sarolaner tem sido utilizado no controle de diversos ectoparasitas, mas não existem registros de seu uso no tratamento de tungíase em cães. O objetivo deste estudo foi avaliar a eficácia do sarolaner no tratamento de cães naturalmente infestados por *Tunga* spp. mantidos no mesmo ambiente infestado. Três dos quatro animais foram medicados com sarolaner por via oral em dose única de 2 mg/kg, conforme recomendação do fabricante, e um animal permaneceu sem medicação. Após 24 horas, as pulgas dos quatro cães foram removidas mecanicamente. Os animais foram reavaliados nos dias +15 e +30 para avaliar uma possível reinfestação. Os animais medicados permaneceram livres de pulgas, enquanto o animal não tratado apresentou pulgas nos dias previamente definidos para reavaliação. Podemos assim concluir que o uso do sarolaner é uma escolha eficaz para o tratamento da tungíase.

## Introduction

Tungiasis is a dermatological parasitic disease caused by the sand flea *Tunga* spp. (Siphonaptera, Tungidae) that affects domestic and wild animals (Jesus, 2023; Santos et al., 2022) and humans (Pampiglione et al., 2009). The dermatopathy is related to the penetration, maturation and thickening of the flea body in the host's skin (Harvey et al., 2021). This ectoparasite is endemic in Latin America and Sub-Saharan Africa (Pampiglione et al., 2009).

Penetration by parasites can variously cause intense inflammation, papular cutaneous lesions with a central dark punctum, swelling, erythema, intense itching, pain, secondary bacterial infection, cellulitis, and necrosis (Eisele et al., 2003; Harvey et al., 2021). The main regions of the body affected are feet, limbs, perineum, mammary gland, gonads, snout, and tail. These are the body regions closest to the soil, where the flea's life cycle occurs outside the host (Harvey et al., 2021; Klimpel et al., 2005; Pampiglione et al., 2009).

Mechanical removal of the flea with the aid of a sterile needle is the main treatment. However, sedation may be necessary (Loft & Nissen, 2009; Schott et al., 2020). Some studies have also reported the usage of drugs to treat animals suffering from *Ctenocephalides felis* infestation, such as permethrin, imidacloprid (Klimpel et al., 2005), ivermectin (Loft & Nissen, 2009) and nitenpyram (Schott et al., 2020), but no consensus exists about the best medical treatment. Isoxazolines are potent long-lasting pulicides that cause uncontrolled neuromuscular activity, leading to rapid insect death (Ozoe et al., 2010; Zhou et al., 2022). Recent studies using fluralaner and afoxolaner have reported excellent efficacy against tungiasis in dogs (Santos et al., 2022, 2023a, 2023b).

The objective of this report is to describe the first treatment of canine tungiasis with a single oral dose of sarolaner at a minimum concentration of 2 mg/kg.

## Cases report

Four healthy mixed-breed dogs were analyzed, with ages between 6 months to 10 years and weighing between 5 and 15 kg, showing normal clinical patterns from the same rural property. All were parasitized by *T*. spp. only on all four paw pads (Table 1). Clinical examination revealed active circular lesions in the form of white patches with central black points, indicating the posterior segments of the enlarged females, which were identified by the Fortaleza classification (Eisele et al., 2003) (stages II and III), leading to the clinical diagnosis of tungiasis.

**Table 1:** Number of *Tunga* spp. fleas in naturally infested dogs submitted to treatment with sarolaner, including one unmediated animal, throughout the experimental period.

Animals	Weight (Kg)	Dose mg/kg	N° of *Tunga penetrans* in paws pads			
			Day 0 Day +1 Day +15 Day +30			
N1*	6,3	3,17	8	0	0	0
N2*	8,1	2,46	8	0	0	0
N3*	9,8	2,04	17	0	0	0
N4**	7,9	-	19	0	12	9

* Animals treated with a minimum dose of 2 mg/kg of sarolaner in a single dose;

** (unmedicated).

Three of four dogs were medicated with a single oral dose of sarolaner (Simparic®, sarolaner, Zoetis, Brazil) at minimum concentration of 2 mg/kg (D0). Twenty-four hours afterward (D+1), the dogs were reassessed, and fleas of all animals were mechanically removed and counted without the need for sedation. We observed involuted lesions in the medicated animals that made it easier to remove their fleas, while in the non-medicated animal, active lesions remained the same, and more effort was required for removal, causing significant bleeding and considerable discomfort. At the end of the procedure, a total of 33 sand fleas were observed in the animals that received the medication (two with eight fleas each and one with 17), while the animal that was treated only with mechanical removal had a total of 19 fleas (Table 1).

A recheck was carried out on the 15^th^ day (D+15) after treatment and the medicated animals remained free of reinfestation, while the dog not treated with saroliner had various sand fleas (stages II and III). Those parasites were again removed mechanically, confirming reinfestation. Reassessments were performed on day 30 (D+30), and the animals that received the medication were free of active lesions from the presence of fleas on the skin, while it was again necessary to mechanically remove sand fleas (stage II) from the non-medicated dog.

## Discussion

Corroborating the report of Harvey et al. (2021), we detected fleas on the paws, with preference for the palmar and plantar paw pads, although the other regions of the body were in frequent contact with the ground. Also in line with Harvey et al. (2021), we found that fleas had preference for more keratinized body regions containing less hair.

Usually, the gold standard treatment is mechanical removal of fleas, which sometimes requires sedation because it is a painful, invasive and stressful procedure (Loft & Nissen, 2009). However, as mentioned above, studies have reported treatment with permethrin, imidacloprid (Klimpel et al., 2005), ivermectin (Loft & Nissen, 2009), nitenpyram (Schott et al., 2020), natural products with topical *psidium guidean* in coconut oil, and even kerosene ointment with naphthalene (Enwemiwe et al., 2020), but so far all are considered off-label for this purpose.

We observed that the previous administration of sarolaner facilitated parasite removal, making it a better option than just removing the sand fleas. Furthermore, we believe that if the mechanical removal had not been carried out, the fleas of all animals that received the medication would probably have detached in a short period of time. This agrees with Santos et al. (2022, 2023b), who found in randomized, negatively controlled and double-blind studies that the use of fluralaner or afoxolaner (drugs also belonging to the isoxazoline group) in a single dose was an excellent therapeutic option against *T.* spp*.*, with respective effectiveness of 90% and 100% for up to 90 days after administration.

According to previous works, in Brazil the prevention and control of tungiasis in animals and humans should be centralized in dogs, since they are considered the main hosts of the parasite in the country. However, the neglect of owners, either by not identifying the lesions or not removing the parasitee, makes the disease difficult to control. Thus, there is a need for broad educational awareness actions among dog owners, stressing the effectve canine therapeutic and prophylactic measures to achieve the One Health concept (Harvey et al., 2021; Jesus, 2023; Santos et al., 2022).

To the best of our knowledge, this is the first report of the treatment of tungiasis in dogs with oral sarolaner, which proved to be an excellent treatment and prevention option, since in subsequent evaluations, only the non-medicated animal was reinfested.

## Conclusion

The use of sarolaner was effective in treating tungiasis in three naturally infested dogs.
